# A novel CXCR4-targeted near-infrared (NIR) fluorescent probe (Peptide R-NIR750) specifically detects CXCR4 expressing tumors

**DOI:** 10.1038/s41598-017-02818-6

**Published:** 2017-05-31

**Authors:** Sara Santagata, Luigi Portella, Maria Napolitano, Adelaide Greco, Crescenzo D’Alterio, Maria Vittoria Barone, Antonio Luciano, Matteo Gramanzini, Luigi Auletta, Claudio Arra, Antonella Zannetti, Stefania Scala

**Affiliations:** 1 Functional Genomics, Istituto Nazionale per lo Studio e la Cura dei Tumori, Fondazione “G. Pascale”-IRCCS, Napoli, Italy; 2 Institute of Biostructure and Bioimaging, National Research Council, Naples, Italy; 3 Department of Advanced Biomedical Science, Federico II University Medical School, Naples, Italy; 4 CEINGE-Advanced Biotechnologies, Naples, Italy; 5 Department of Translational Medical Science and European Laboratory for the Investigation of Food Induced Disease (ELFID), University of Naples, Federico II, Naples, Italy; 6 Animal Facility Unit, Istituto Nazionale per lo Studio e la Cura dei Tumori, Fondazione “G. Pascale”-IRCCS, Napoli, Italy; 7 IRCCS SDN, Naples, Italy

## Abstract

C-X-C chemokine receptor 4 (CXCR4) is over-expressed in multiple human cancers and correlates with tumor aggressiveness, poor prognosis and increased risk for distant metastases. Imaging agents for CXCR4 are thus highly desirable. We developed a novel CXCR4-targeted near-infrared (NIR) fluorescent probe (Peptide R-NIR750) conjugating the new developed CXCR4 peptidic antagonist Peptide R with the NIR fluorescent dye VivoTag-S750. Specific CXCR4 binding was obtained in cells overexpressing human CXCR4 (B16-hCXCR4 and human melanoma cells PES43), but not in CXCR4 low expressing cells (FB-1). *Ex vivo* evaluation demonstrated that PepR-NIR750 specifically detects B16-hCXCR4-derived subcutaneous tumors and lung metastases. Fluorescence Molecular Tomography (FMT) *in vivo* imaging was performed on mice carrying subcutaneous CHO and CHO-CXCR4 tumors. PepR-NIR750 accumulates only in CXCR4-positive expressing subcutaneous tumors. Additionally, an intense NIR fluorescence signal was detected in PES43-derived lung metastases of nude mice injected with PepR-NIR750 versus mice injected with VivoTag-S750. With a therapeutic intent, mice bearing PES43-derived lung metastases were treated with Peptide R. A the dramatic reduction in PES43-derived lung metastases was detected through a decrease of the PepR-NIR750 signal. PepR-NIR750 is a specific probe for non-invasive detection of human high CXCR4-expressing tumors and metastatic lesion and thus a valuable tool for cancer molecular imaging.

## Introduction

Current imaging methods for early cancer detection are limited by low specificity and sensitivity^1^. Optical imaging offer promising noninvasive, real-time and high-resolution modalities^2^ and Near-infrared (NIR) fluorescence probes, that emit in the NIR region (650–900 nm), are characterized by low auto fluorescence and deep tissue penetration with minimal background interference representing ideal candidates for cancer targeted imaging^3, 4^.

The chemokine receptor 4 (CXCR4) is a G-protein-coupled receptor devoted to regulate leukocyte trafficking^5^; it is expressed in 23 different cancers where it plays a critical role in tumor progression and metastatic spread^6^. High CXCR4 expression characterize cancer cells with high migratory capability and biological aggressiveness^7^. A recent meta-analysis considering 85 studies in more than 11,000 patients with cancer revealed that over-expression of CXCR4 associates with worse prognosis in terms of overall survival (OS) and progression-free survival (PFS) in different types of tumors^8^. The CXCR4 ligand CXCL12 is mainly expressed by mesenchymal stromal cells in liver, lungs and bone marrow (BM) where CXCR4-positive cancer cells can be recruited to initiate metastasis^9^. Recently, we demonstrated that bone marrow mesenchymal stem cells (BM-MSCs) induce osteosarcoma and hepatocellular carcinoma progression through CXCR4 activation^10^. Furthermore, CXCL12 can attract CXCR4-positive immune cells or fibroblasts to the tumor sites to assist in tumor development. High CXCL12 in tumors attract CXCR4-positive inflammatory, vascular and stromal cells that support tumor by secreting growth factors, cytokines, chemokines and pro-angiogenic factors^9^. CXCR4 is also expressed on normal stem cells^11^ and in prostate and pancreatic cancer progenitors^12, 13^. Recently, we reported that CXCR4 and CD133 expression identified a discrete population with stem cell properties in human ovarian cancer cells that might be critical for tumor development and chemo-resistance^14^. Thus early detection of CXCR4 positive cancer cells may identify and target an aggressive cellular cancer component^15^. A new class of rationally designed CXCR4 cyclic peptide antagonists was recently developed by us. Three novel peptides impaired CXCR4 function *in vitro* and *in vivo* with peptide R being the most efficient in “*in vivo*” studies^16^. With the intent to develop a CXCR4-targeted NIR fluorescent imaging agent Peptide R was labelled with Vivo Tag-S 750 dye (Peptide R-NIR750). CXCR4 selectivity and sensitivity was evaluated in *in vitro* and *in vivo* CXCR4 primary and secondary tumors. Furthermore, Peptide R was developed as anti-metastatic agents and its potential of theranostic agent in cancer was demonstrated.

## Results

### PepR-NIR750 specifically binds CXCR4 expressing cancer cells

**Figure 1:**
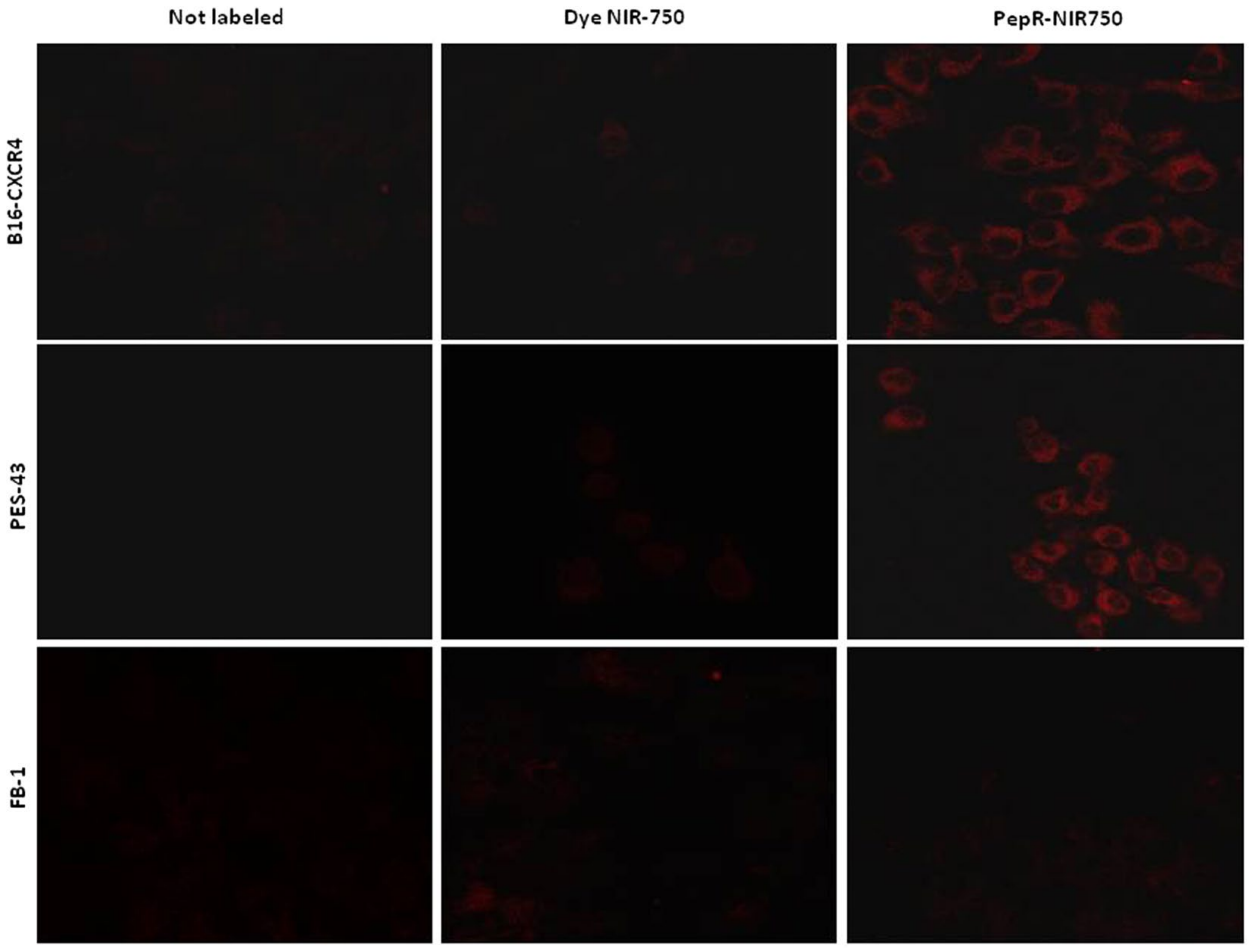
PepR-NIR750 specifically binds CXCR4 expressing cells. B16-CXCR4 and PES43 tumor cell lines expressing high CXCR4 levels and FB1 cells expressing very low CXCR4 levels were plated on glass coverslips and labeled with PepR-NIR750 or VivoTag-S 750 alone (100 nM) for 1 h at 37 °C; nuclei were labeled with DAPI and the fluorescence was observed by confocal microscope (LSM 510 Zeiss). PepR-NIR750 binds to B16-CXCR4 and PES43 cells but not to FB-1 cells whereas VivoTag-S 750 signal is not detected in all cell lines tested.

Peptide R was conjugated with Vivo-Tag S750 NIR-dye according to the manufacture instruction (see methods section) (PepR-NIR750). The ability of PepR-NIR750 to bind CXCR4 was evaluated on cancer cell lines differentially expressing the receptor: FB1, human anaplastic thyroid cancer cells, known to express low level of CXCR4; CHO, Chinese hamster ovarian cells and CHO cells transfected with human CXCR4; PES43, human melanoma cell line and B16 mouse melanoma cell lines transfected with human CXCR4 (B16-CXCR4) (Figure S1)^17, 18^. As shown in Fig. 1, PepR-NIR750 binds CXCR4 on B16-CXCR4 and PES43 cells but not on FB-1 cells. No signal was detected in the presence of the dye VivoTag-S 750 alone, demonstrating the specificity of PepR-NIR750 in visualizing CXCR4 expressing cells.

### *In vivo* binding of CXCR4 expressing subcutaneous tumor and lung metastasis by PepR-NIR750

**Figure 2:**
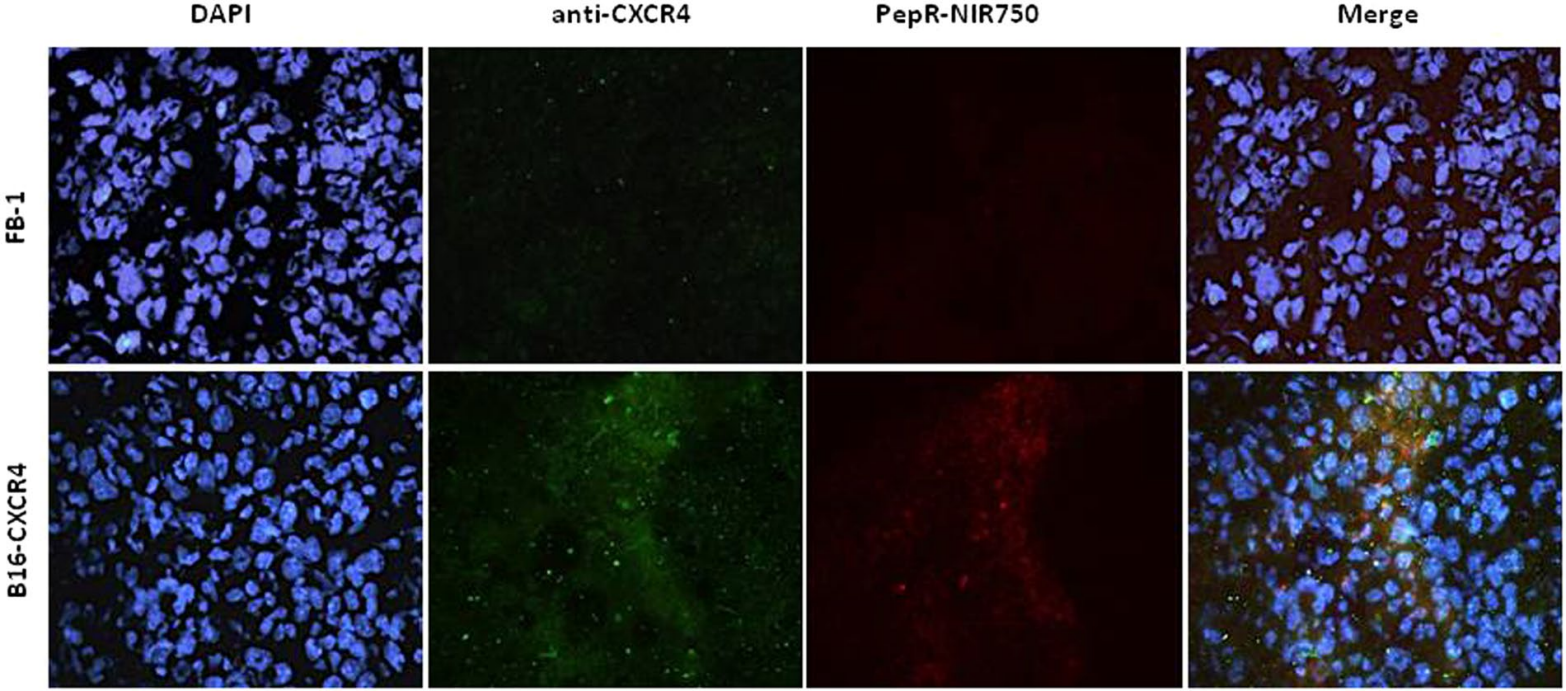
*In vivo* evaluation of PepR-NIR750 binding to CXCR4 expressing subcutaneous tumors. B16-CXCR4 and FB-1 subcutaneous tumors in CD-1 nu/nu athymic mice were *i*.*v*. injected with PepR-NIR750 (red) and examined with a fluorescent CXCR4 antibody (green) and imaged at fluorescence microscope Axioscope A.1. Nuclei were stained with DAPI. Merged images of anti-CXCR4 antibody staining and PepR-NIR750 fluorescence are shown in the last panel. PepR-NIR750 binds to B16-CXCR4 cells but not to FB-1 cells.

**Figure 3:**
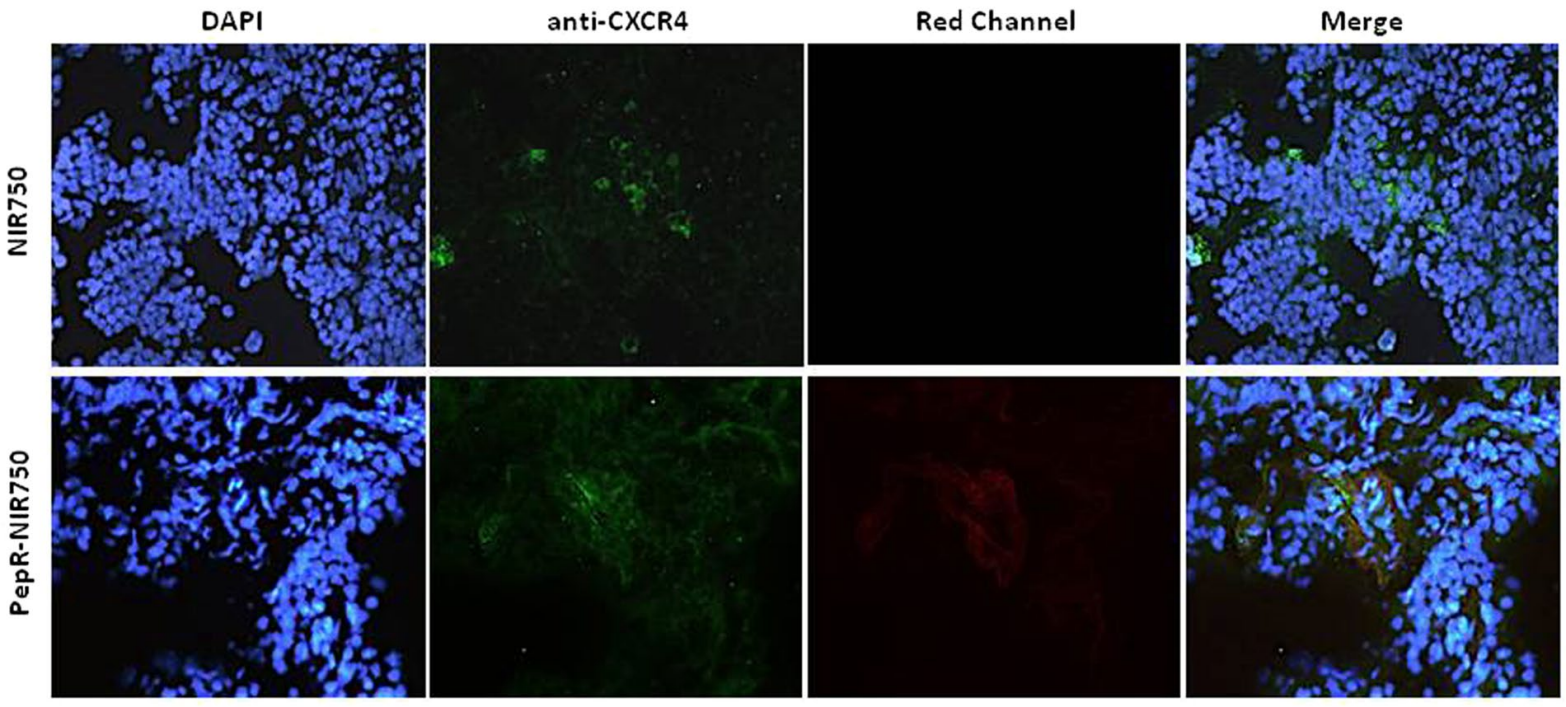
*In vivo* evaluation of PepR-NIR750 binding to B16-CXCR4 lung metastasis. B16-CXCR4 syngenic tumor model of lung metastases, were obtained by *i*.*v*. injection of B16-CXCR4 of CD-1 nu/nu athymic mice. After 3 weeks mice were *i*.*v*. injected with PepR-NIR750 (5 nM) or VivoTag-S 750 alone and lungs were stained with a fluorescent CXCR4 antibody (green) and analyzed for PepR-NIR750 (red) staining. Sections obtained from PepR-NIR750 or VivoTag-S 750 -injected mice lungs were imaged at fluorescence microscope Axioscope A.1. Nuclei were stained with DAPI. Merged images of anti-CXCR4 antibody staining and PepR-NIR750 or VivoTag-S 750 alone fluorescence are shown in the last panel. The PepR-NIR750 signal is detected in lung metastasis and co-localizes with anti-CXCR4 staining.

To evaluate PepR-NIR750 capacity to bind CXCR4 expressing cells *in vivo*, FB-1 cells and B16-CXCR4 cells were *s*.*c*. inoculated in CD-1 nu/nu athymic mice. When tumors reached 100 mm^3^, mice were *i*.*v*. injected with PepR-NIR750 and after 1 hour of tracer biodistribution mice were euthanized and tumors explanted. Tumor sections were stained with a-CXCR4/AlexaFluor488 and DAPI. As shown in Fig. 2, PepR-NIR750 signal (red) was detected in B16-CXCR4 tumors but not in FB-1 tumors. B16-CXCR4 and FB-1 tumors were respectively positive and negative for CXCR4 staining (green). CXCR4/AlexaFluor488 and PepR-NIR750 staining overlapped in B16-CXCR4 tumor sections confirming the same target for PepR-NIR750 and CXCR4/AlexaFluor488. In addition, B16-CXCR4 (350,000 cells/mice) were *i*.*v*. inoculated in 6 mice. Three weeks later the mice were injected with PepR-NIR750 and lung metastasis evaluated for specific PepR-NIR750 versus VivoTag-S 750 staining. As shown in Fig. 3 PepR-NIR750 (red) labelled B16-CXCR4 derived lung metastases and co-localized with CXCR4 (green) in metastatic cells. Control probe, VivoTag-S 750 did not label CXCR4-expressing metastatic cells. Thus PepR-NIR750 specifically binds to CXCR4-expressing cells *in vivo*.

### FMT *in vivo* imaging of CHO-CXCR4 subcutaneous tumors by PepR-NIR750

**Figure 4:**
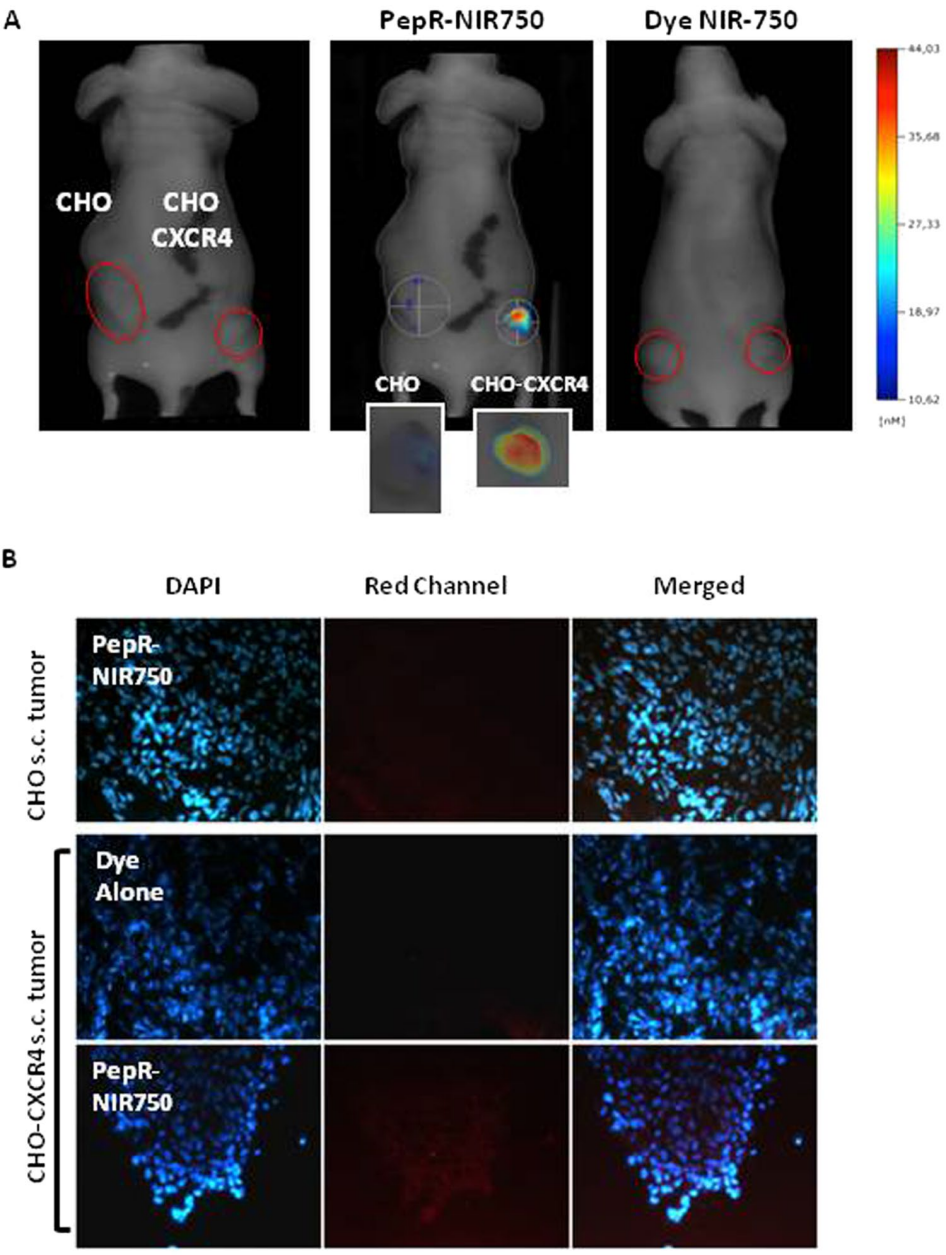
*In vivo* imaging of CXCR4 expressing tumors by PepR-NIR750. Imaging studies were carried out 1 hour after injection of NIR probe in CHO and CHO-CXCR4-derived *s*.*c*. tumor models. (**A**) 5 nM PepR-NIR750 or VivoTag-S 750 alone was *i*.*v*. injected and total body NIR fluorescence was monitored using fluorescence molecular tomography FMT4000. PepR-NIR750 detects CHO-CXCR4 tumor but not CHO tumor. No NIR fluorescence signal was detected in *s*.*c*. tumors from mouse injected with VivoTag-S 750 alone. (**A**, smaller panels) After imaging study mice were euthanized and tumors explanted were observed by FMT4000. (**B**) 5 µm sliced obtained from snap frozen tumors were stained with DAPI and analyzed for NIR fluorescence signals (red) by fluorescence microscope Axioscope A.1.

CHO and CHO-CXCR4 cells were *s*.*c*. injected respectively in the left and the right flank of CD-1 nu/nu athymic mice. When the average size of tumors reached approximately 100 mm^3^, mice were injected *i*.*v*. with PepR-NIR750 or VivoTag-S 750 alone. FMT4000 imaging showed that PepR-NIR750 detects CHO-CXCR4 tumor but not CHO tumor. VivoTag-S 750 alone failed to visualize tumors (Fig. 4A). As shown in Fig. 4A,B the explanted tumors evaluated by FMT (4A, smaller panels) and fluorescence microscopy (4B) confirmed the specific binding of PepR-NIR750 to CHO-CXCR4 tumors.

### FMT *in vivo* imaging of CXCR4 expressing human melanoma lung metastasis by PepR-NIR750

**Figure 5:**
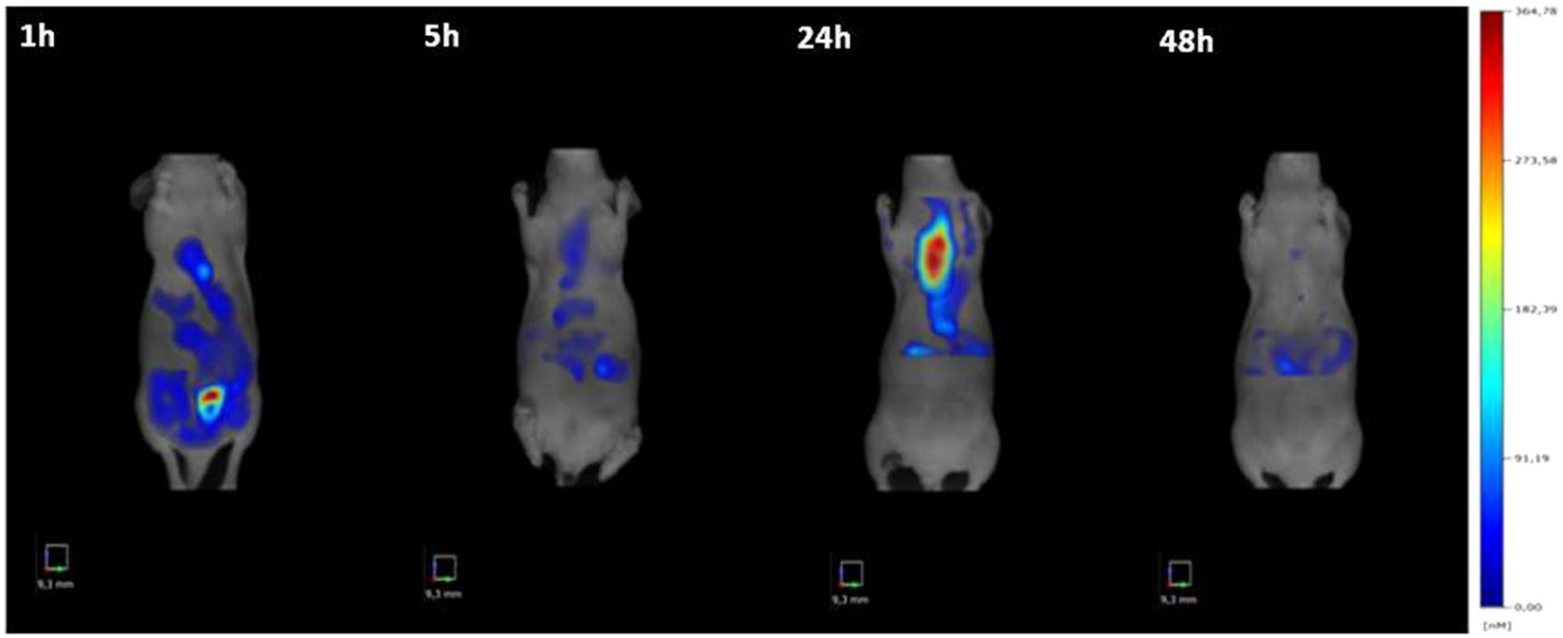
*In vivo* imaging of CXCR4 expressing human melanoma lung metastasis by PepR-NIR750. Mice bearing PES43-derived lung metastases were *i*.*v*. injected with Pep-R-NIR750 and imaged 3 weeks after cells inoculation. Total body imaging studies were carried out 1 h–5 h–24 h–48 h after tracer injection with FMT 4000. The NIR image shows a strong signal of PepR-NIR750 in the lungs after 24 hours from injection.

PES43, human melanoma cells, were *i.v.* injected (2 × 10^6^) and three weeks later developed lung metastases in CD-1 nu/nu athymic mice. With the intent to verify the PepR-NIR750 imaging, mice were *i*.*v*. injected with PepR-NIR750 and imaged at different time points (1–5–24–48 hours) by FMT4000. As shown in Fig. 5, PepR-NIR750 uptake in metastatic lungs reached a peak of fluorescence intensity at 24 hours and declined at 48 hours post injection. The whole body PepR-NIR750 biodistribution showed no significant uptake in the normal mouse organs.

### PepR-NIR750 imaging detects lung metastases reduction induced by Peptide R treatment

**Figure 6:**
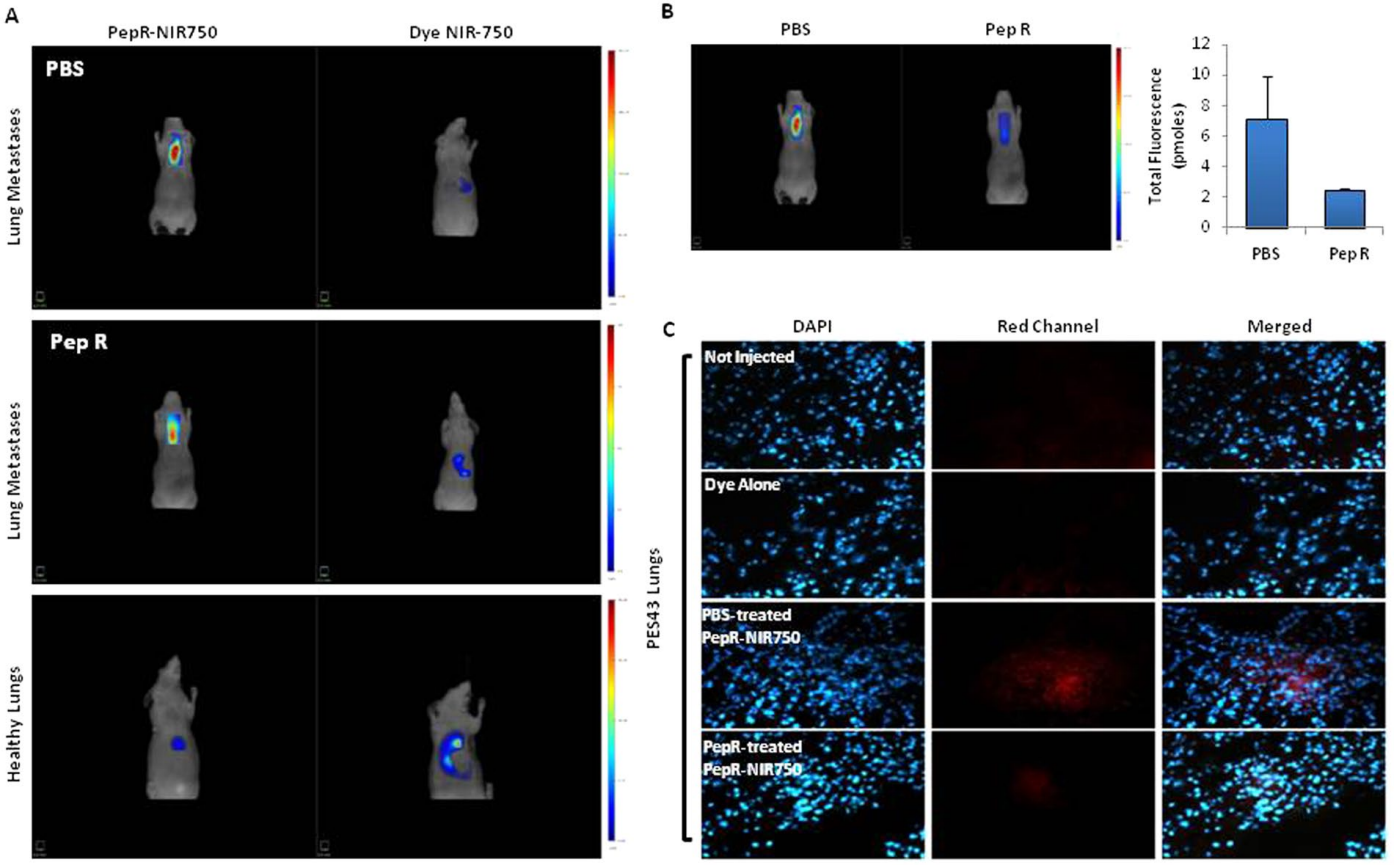
PepR-NIR750 detects lung metastases reduction induced by Peptide R treatment. CD-1 nu/nu athymic mice bearing PES43-derived lung metastases were treated with PBS (control group) or Peptide R (2 mg/Kg) 5 days/week for 2 weeks. 3 weeks later mice were *i*.*v*. injected with PepR-NIR750 or VivoTag-S 750 alone and total body imaged after 24 h with FMT4000. (**A**) PepR-NIR750 signal is detectable in mice bearing lung metastases (upper and middle left panels) while no signal was revealed with VivoTag-S 750 alone (upper and middle right panels). Healthy lungs are not imaged by PepR-NIR750 (lower panel). (**B**) *In vivo* imaging of mice bearing lung metastases treated and not treated with Peptide R using Pep-R-NIR750. Peptide R causes a strong reduction of PepR-NIR750 signal (65%) respect to control group as observed 24 hours after its injection. (**C**) After 24 h the mice were euthanized, the lungs snap frozen and then were 5 µm sliced at cryostat microtome. Slides of PepR-NIR750 or VivoTag-S 750 were imaged at fluorescence microscope Axioscope A.1.

**Figure 7:**
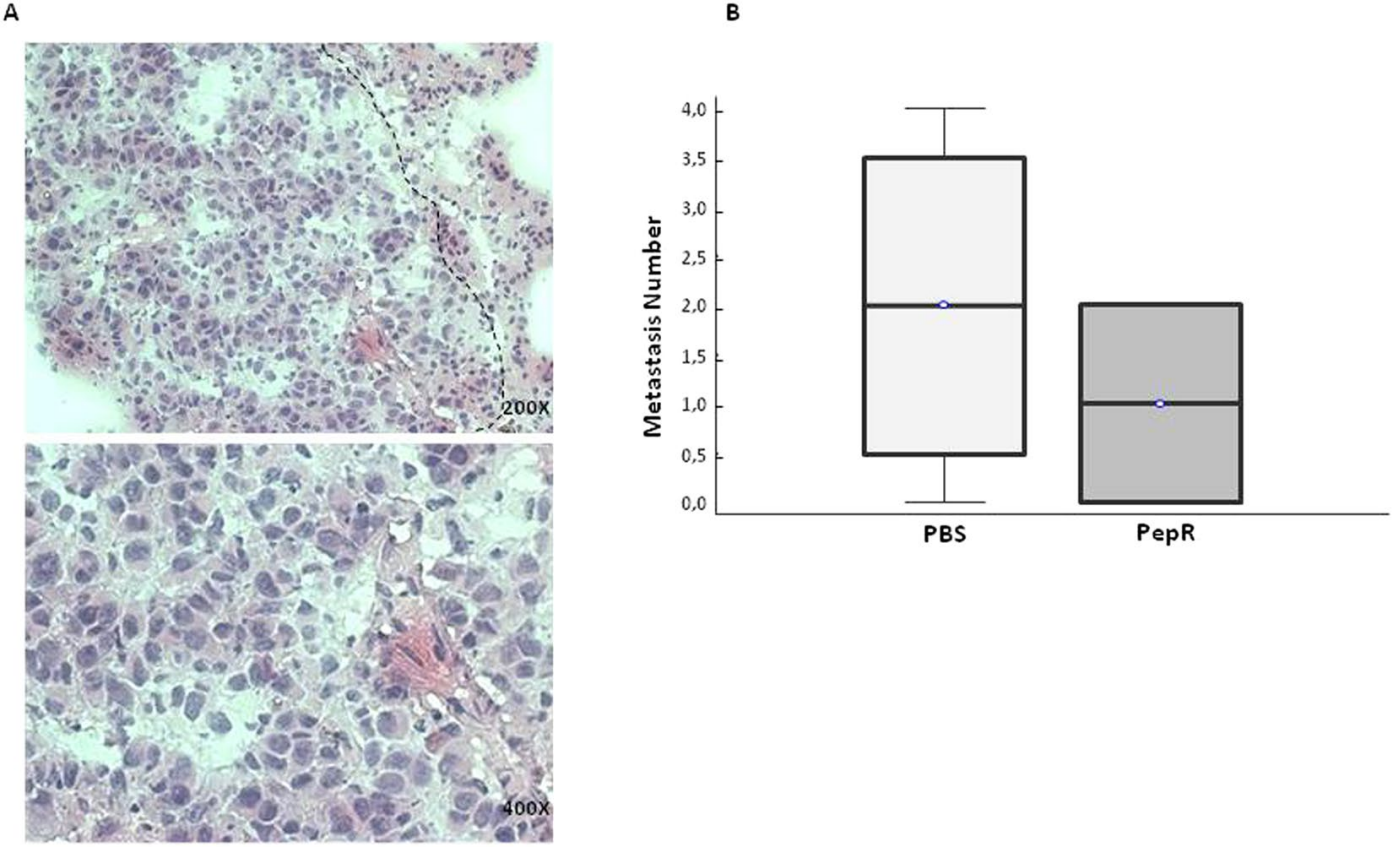
PES-43 lung metastasis detection and quantitation in untreated mice vs Pep-R treated mice. (**A**) Representative microphotographs of PES43 metastatic nodule, H&E stained 5 micron-thickness tissue slide, using a cryostat frozen OCT embedded lung (200× magnification). (**B**) Box and Whisker plot showing median, upper/lower quartile and min/max of pulmonary metastatic nodules. H&E evaluation confirmed a presence of lung metastasis in control group and a decrease of metastasis number in mice treated with Peptide R compared to control group.

Peptide R treatment was previously shown to inhibit the development of lung metastases in mice^16^. To evaluate the capability of Peptide R to reduce the development of metastatic lung lesions, human melanoma cells PES43 cells were *i*.*v*. injected in nude mice and the day after treatment with Peptide R started. Peptide R (2 mg/Kg) or PBS (control) were *i*.*p*. administrated once a day for 10 days. Three weeks later mice were *i*.*v*. inoculated with PepR-NIR750 and visualized by FMT4000. As shown in Fig. 6A an intense NIR fluorescence signal was detected in lung metastases 24 hours post-injection of PepR-NIR750, while no signal was revealed in mice injected with VivoTag-S 750 alone. In addition, PepR-NIR750 signal was not detectable in healthy lungs. Peptide R treatment determined a dramatic reduction of PepR-NIR750 signal (65%) mainly detected 24 hours after injection (Fig. 6B). These evidence were confirmed by the *ex vivo* study conducted on lung sections (Fig. 6C). As expected, tissue analysis confirmed the presence of lung metastases (Fig. 7A) and their reduction after Peptide R treatment (Fig. 7B).

## Discussion

A novel CXCR4 targeted NIR fluorescent imaging agent (Peptide R-NIR750) was described. Peptide R belongs to a new family of CXCR4 peptides developed and characterized as potent CXCR4 antagonists with anti-metastatic potential in *in vivo* models^16^. CXCR4 antagonist peptide binds tumor cells overexpressing the receptor; in addition CXCR4 modulation regulates the access of T effector cells to tumor microenvironment affecting the efficacy of immunotherapy^19, 20^. With the intent to magnify the checkpoints inhibitors (CI) response ongoing clinical trials couple CXCR4 antagonists to CI (NCT02823405, NCT02680782, NCT02737072, NCT02826486 NCT02472977). The PepR-NIR750 CXCR4 tracer will allow to visualize *in vivo* the CXCR4 modulating drugs on tumor microenvironment through labeling of CXCR4 positive T and immunoregulatory cells. BM-MSCs (bone marrow-mesenchymal cells) are recruited to the tumor microenvironment contributing to build the metastatic niches. We previously showed that BM-MSCs potentiate osteosarcoma and hepatocellular carcinoma cells growth and migration and that treatment with CXCR4 antagonists, AMD3100 and Peptide R, reduced migration and invasion BM-MSCs mediated^10^. Here we show that Peptide R-NIR750 specifically binds to CXCR4 overexpressing tumor cells *in vitro* and *in vivo* and discriminates the effect of Peptide R treatment on the development of lung metastases through fluorescence molecular tomography. Fluorescence molecular tomography has been demonstrated to detect and quantify fluorescent biomarkers in sites of cancer, inflammation, and infectious disease. This technology allows few centimeters deep imaging, high sensitivity, low background and is radioactive-free^21^. A novel peptide CXCR4-targeted-NIR fluorescence imaging agent (CXCR4-IR-783) was recently described to detect primary osteosarcoma and lung micro-metastasis^22^ although the authors are seeking improvements of the probe aimed to reduce the high signal-to-noise ratio and improve specificity toward neoplastic cells. Our tracer conjugates a new peptide CXCR4 antagonist belonging to a rational-designed patented new family of CXCR4 antagonists (PCT/IB2011/000120/EP 2 528 936 B1/US2013/0079292A1) to the near infrared probe. Peptide R itself has an inhibitory effect on CXCR4 coupling double effect of labeling and inhibiting the CXCR4 receptor^16^.

In addition, Meinke *et al*. synthesized conjugates of the CXCR4 ligand, CXCL12, with the NIR fluorescent dye IRDye 800CW to visualizes breast and glioma xenografts through CXCR4 and CXCR7 targeting^23^. The unique CXCR4 antagonist clinically approved is AMD3100 indicated for hematopoietic stem cell mobilization^24^. Other receptors are also druggable to induce hematopoietic stem cell mobilization. Targeting α9β1/α4β1 integrins with a single dose of a small molecule antagonist (BOP (N-(benzenesulfonyl)-L-prolyl-L-O-(1-pyrrolidinylcarbonyl)tyrosine) rapidly mobilizes long-term multi-lineage reconstituting hematopoietic stem cells (HSC); nevertheless synergistic engraftment is observed when BOP is co-administered with AMD3100. The authors, with a related fluorescent analogue of BOP (R-BC154), showed that this class of antagonists preferentially bind human and mouse HSC and progenitors within the endosteal niche^25^. PepR-NIR750 will bind hematopioeic precursors *in vivo* allowing to follow and understand the mobilization process as again, Peptide R itself was demonstrated to mobilize mouse hematopoietic precursor cells (Portella L. *et al*. AACR 2011; number 394). We are currently evaluating Peptide R-NIR750 to study how the receptor dynamic and CXCR4 transport is regulated in the presence of CXCR4 antagonists. Moreover the recruitment and retention of progenitor cells in ischemic tissue are regulated by CXCR4 and hypoxia^26, 27^; thus tracing migration of endothelial precursors through Peptide R-NIR750 is feasible and may add insights into the CXCR4 antagonists mechanism of action. It was previously demonstrated that Peptide R inhibits recruitment of intratumoral bone marrow derived cells in B16-hCXCR4-lung metastases in CXCR4−/+ mice. A reduction in LY6G-positive myeloid/granulocytic cells and in p38 MAPK activation was detected in lungs from CXCR4(+/−) mice compared to CXCR4(+/+) mice suggesting that CXCR4 reduction on myeloid-derived cells decreased their recruitment to the lung, consequently impairing lung metastases^28^. In ongoing studies the Peptide R-NIR750 traces noninvasively BMDC migration.

In conclusion, the fluorescent probe Peptide R-NIR750 is a theranostic tool specifically targeting CXCR4 expressing cells in *in vivo* models. Peptide R has a therapeutic efficacy in preventing the development of lung metastases. This tool will allow pharmacodynamics and mechanistic studies on CXCR4 antagonists on neoplastic cells and immune cells shedding further insights and optimizing therapies targeting CXCR4 alone and in combination with immunomodulatory drugs.

## Materials and Methods

### Peptide conjugation and purification

Cyclic peptide R was synthesized on solid phase by using Fmoc chemistry standard protocols as previously described^16^ (Supplementary Fig. S2A). VivoTag-S 750-*N*-hydroxysuccinumide (NHS) ester was purchased from PerkinElmer, Inc, Boston, MA, USA. Peptide R (1 nmol) was incubated in 50 µl 0.1 M Na2CO3 buffer, pH 8.6, with 5 nmol VivoTag-S 750-*N*-hydroxysuccinumide (NHS) ester dissolved in 5 µl dimethyl sulfoxide for 2 h at room temperature in the dark. Excess reactive groups were saturated by addition of 2 µl 1 M Tris–HCl buffer, pH 8.6, and incubate for additional 15 min. Then, conjugates were separated on Zeba Spin Desalting Columns (Thermo Scientific, Rockford, IL61105 USA) and eluted with 250 µl 0.14 M NaCl in 20 mM Hepes buffer, pH 7.4, under centrifugation (2 min, 1000 × *g*) (Supplementary Fig. S2B).

### Cell culture

Tumor cell lines were grown in appropriate medium with 10% fetal bovine serum (FBS) 2 mM glutamine, 50 mg/mL penicillin, 50 mg/mL streptomycin (complete medium) at 37 °C in 5%CO2. PES43 human melanoma cells derived from a lung metastases of melanoma patient were grown in Iscove’s Modified Dulbecco’s Medium (IMDM). FB-1 human anaplastic thyroid cancer cell line (kindly provided by Dr. Melillo, University of Naples, Federico II, Italy) were grown in Dulbecco’s Modified Eagle’s Medium (DMED). B16-CXCR4 murine melanoma cells were obtained by transfection of B16 with pYF1-fusin plasmid containing human CXCR4 gene (kindly provided by Dr Aloj, NCI “Pascale”, Naples, Italy) using Fugen 6 (Roche Applied Science, Indianapolis, IN) in according to manufacturer’s instruction and grown in complete medium supplemented with 100 µg/mL G418. CHO and CHO cells transfected with human CXCR4 (CHO-CXCR4) were kindly provided by Dr. David McDermott (NIH, Bethesda, USA) and grown in complete medium supplemented with 1 mg/mL G418.

### Immunofluorescence microscopy

For *in vitro* binding experiments, cells were seeded (1 × 10^4^ cells/well) on coverslips in 24-well plates in 1,5 ml growth complete medium. After five days the medium was replaced with 1 ml of fresh medium and the cells were incubated for 1 hour at 37 °C with Peptide R-VivoTag-S 750 (PepR-NIR750) conjugate or VivoTag-S 750 alone at 100 nM concentration. Cells were washed three times with PBS, and incubated in 4% BSA in this buffer. Cells were fixed with 4% paraformaldehyde for 15 min at room temperature (RT) and DAPI nuclei staining performed. The coverslips were then mounted on glass slides and fluorescence was observed by confocal microscope (LSM 510 Zeiss). CXCR4 immunofluorescence was conducted on tumor derived frozen tissues with a CXCR4 antibody clone 44716 (R&D Biosystem) and anti-mouse AlexaFluor488 (Jackson) according to manufacture instruction. Slides were imaged at fluorescence microscope Axioscope A.1 equipped with proper optical filter.

### Mouse Tumor Models

All experimental procedures complied with the European Communities Council directives (2010/63/EU) and national regulations (D.L. 116/92) and were performed in accordance with National Institutes of Health (NIH) recommendations. The present study was approved by the Italian Ministry of Health (authorization number 2013/0100808). All efforts were made to minimize animal suffering and the number of animals necessary to produce reliable results. All experimental procedures described were performed under general anesthesia with 2% isoflurane in 100% oxygen at 0.8 L/min. Subcutaneous models were realized with 3 × 10^5^ B16-CXCR4 cells and 10 × 10^6^ FB-1 cells, respectively, injected in the flank of CD-1 nu/nu athymic mice. Alternatively, 10 × 10^6^ CHO and CHO-CXCR4 cells were injected in right and left flank of CD-1 nu/nu athymic mice. Once tumors became palpable (established), approximately 100 mm^3^ [volume = 0.5 × long diameter × (short diameter)^2^], nude mice were *i*.*v*. injected with PepR-NIR750 (5 nM) or VivoTag-S 750 alone. B16-CXCR4 syngenic tumor model of lung metastases were realized with 3,5 × 10^5^ B16-CXCR4 injected in the tail vein of 6 CD-1 nu/nu athymic mice. After 3 weeks mice were divided in 2 groups (3 mice groups), *i*.*v*. injected with PepR-NIR750 (5 nM) or VivoTag-S 750 alone and sacrificed after 1 hour. To obtain human model of lung metastases 8 CD-1 nu/nu athymic mice were injected in the tail vein with 2 × 10^6^ PES43 human melanoma cells and treated with PBS or Peptide R (2 mg/Kg) 5 days/week for 2 weeks; after 3 weeks mice were *i*.*v*. injected with PepR-NIR750 or VivoTag-S 750 alone.

### Fluorescence tomography (FMT)

For FMT4000 Quantitative Tomography Imaging *In Vivo* Imaging System (PerkinElmer, Inc.) studies, tumor-bearing mice were maintained on a diet with a purified, alfalfa-free rodent chow for 15 days before fluorescent imaging to minimize fluorescence in the gut. Mice were placed in a biplanar imaging cassette supplied with the instrument and trans illuminated with laser light. Resulting transmission and fluorescence patterns were captured with a thermoelectrically cooled CCD camera, and the position and intensity of fluorescence sources were reconstructed in 3D using the TrueQuant software package (Perkin Elmer, Inc.), supplied with the FMT4000. 3D regions of interest (ROIs) were drawn around tumor regions, and a threshold was applied equal to 30% of the maximum value of fluorescence in the adjoining non-tumor area. The total amount (pmoles) of fluorochrome was automatically calculated relative to internal standards generated with known concentrations of the appropriate dye. After imaging studies mice were euthanized.

### Statistical analysis

All data were presented as mean ± SD. Significance of difference was analyzed with a two-tailed Student’s t test and p < 0.05 were considered statistically significant. The metastasis number was compared among two groups of mice (control group and Peptide R treated group) using Kruskal-Wallis test.

## Electronic supplementary material

Supplementary figures and figure legends
