# Supplementary material for: A novel CXCR4-targeted near-infrared (NIR) fluorescent probe (Peptide R-NIR750) specifically detects CXCR4 expressing tumors
Source: Sci Rep. 2017 May 31;7:2554. doi: 10.1038/s41598-017-02818-6 (PMC5451476; doi:10.1038/s41598-017-02818-6)
Supplement: Supplementary file 1 — Supplementary figures and figure legends [file 41598_2017_2818_MOESM1_ESM.pdf]

A novel CXCR4-targeted near-infrared (NIR) fluorescent probe (Peptide R-NIR750) specifically detects CXCR4 expressing tumors.

Sara Santagata[1], Luigi Portella[1], Maria Napolitano[1], Adelaide Greco[2,3,4], Crescenzo D'Alterio[1], Maria Vittoria Barone[5], Antonio Luciano[6], Matteo Gramanzini[2,4], Luigi Auletta[7], Claudio Arra[6], Antonella Zannetti[2,4#*] and Stefania Scala[1#*]

[1]Functional Genomics, Istituto Nazionale per lo Studio e la Cura dei Tumori, Fondazione "G. Pascale"-IRCCS-Napoli , Italy.

[2] Institute of Biostructure and Bioimaging, National Research Council, Naples, Italy.

[3]Department of Advanced Biomedical Science, Federico II University Medical School, Naples, Italy.

[4]CEINGE-Advanced Biotechnologies, Naples, Italy;

[5]Department of Translational Medical Science and European Laboratory for the Investigation of Food Induced Disease (ELFID), University of Naples, Federico II, Via S. Pansini 5, 80131, Naples, Italy.

[6]Animal Facility Unit, Istituto Nazionale per lo Studio e la Cura dei Tumori, Fondazione "G. Pascale"-IRCCS-Napoli, Italy.

[7]IRCCS SDN, Naples, Italy.

[#] A. Zannetti and S. Scala contributed equally to this work

Supplementary Figure S1

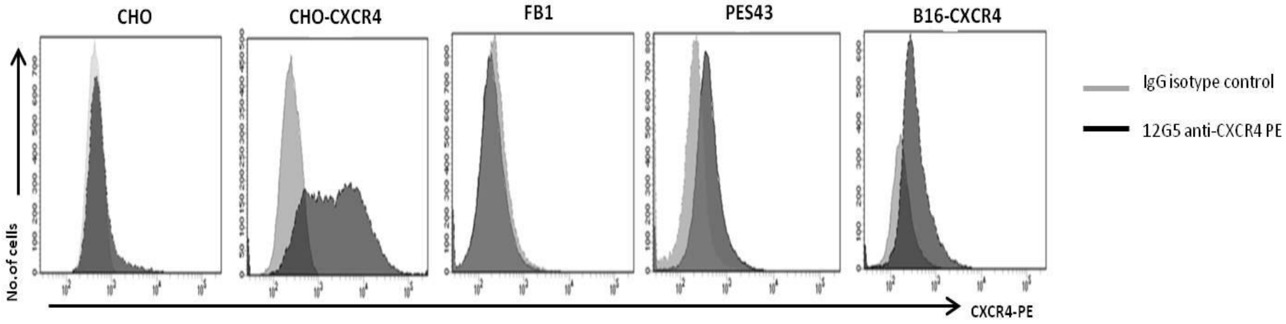

Cell Lines	CXCR4 (%) (12G5 anti-CXCR4 PE)
CHO	0.9
CHO-CXCR4	82
FB1	5,6
PES43	69
B16-CXCR4	79

Supplementary Figure S1 legend

Figure S1. CXCR4 profile in CHO, CHO-human CXCR4, FB1, PES43 and B16 -human CXCR4 cells through flow cytometry. Cells were incubated with the phycoerythrin (PE) conjugated anti-hCXCR4 (clone 12G5).

Supplementary Figure S2

A

Peptide R

Arg-Ala-[Cys-Arg-Phe-Phe-Cys]

B

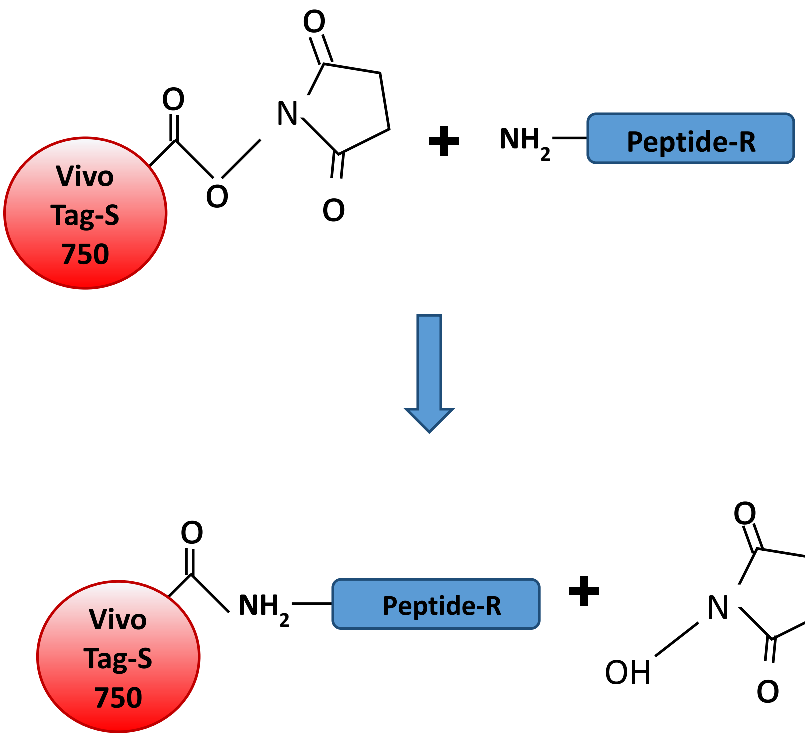

Supplementary Figure S2 legend

Figure S2. (A) Amino acid composition of CXCR4 peptide; (B) Illustration of Peptide R and VivoTag-S 750-N-hydroxysuccinumide (NHS) ester conjugation.
